# Parental migration and disruptions in everyday life: reactions of left-behind children in Southeast Asia

**DOI:** 10.1080/1369183X.2018.1547022

**Published:** 2019-01-04

**Authors:** Theodora Lam, Brenda S. A. Yeoh

**Affiliations:** a Asia Research Institute, National University of Singapore, Singapore; b Department of Geography and Asia Research Institute, National University of Singapore, Singapore

**Keywords:** Left-behind children, children’s agency, transnational migration, web of care, Indonesia, Philippines

## Abstract

Increasing feminisation of transnational labour migration has raised concerns over potential ‘care crises’ at home, and consequently a ‘care deficit’ for children left in origin countries. Our paper focuses on how left-behind children from Indonesia and the Philippines understand, engage and react to changes in their everyday lives in their parents’ absence. While many children had no say over their care arrangements, some were able to assert their agency in influencing their parents’ decisions and eventually migratory behaviours. Their thoughts and actions reinforce the importance of including children’s views in development and migration studies to improve both the children’s and families’ well-being, and make migration a sustainable strategy for all.

## Introduction

Migration, though an important livelihood strategy for many families in Southeast Asia, is also ‘inherently characterized by rupture – a break, change, distance, division’ in everyday life (Boehm et al. 2011, 1). Parental absence in particular is known to cause some ‘displacement, disruptions and changes in caregiving arrangements’ in the family (ECMI/AOS-Manila, SMC & OWWA 2004, 61). However, the experiences, feelings and reactions of children affected by migration are often hidden within a predominantly adult-centric literature on migration despite the fact that children are often situated at the heart of migration decisions made largely for their sakes. This article shifts the focus onto the experiences of Indonesian and Filipino children left by migrant parents in their home countries. It questions how left-behind children from these two countries understand, engage and react to the various disruptions in their everyday life, particularly care arrangements, brought about by their parents’ migration. Pivotal to these explorations is the attempt to highlight and understand children’s agency, resilience and creativity in navigating parental migration (Huang and Yeoh 2011).

Using both quantitative and qualitative data from a mixed-method study on child health and migrant parents in Southeast Asia (CHAMPSEA), our paper first examines childhood in the context of Indonesia and Philippines, and children’s experiences at different stages of the migration process – from being left behind by migrant parent(s) and being cared for by other family and/or non-family members, to reuniting with their returned-migrant parent(s) after their overseas work stints. To further explore how migration affects children’s experiences of ‘family life cycle’ and ‘family care regimes’ (Bryceson 2019), it considers children’s influence on family migration decision-making, their sensitivity to their parents’ absence as well as their responses to the evolving care arrangements necessitated by parental absences. A final section investigates the implications of parental migration for the well-being of left-behind children, and the ways childhood may have been interrupted or reconstructed for the many left-behind children.

## Growing up: observations from Indonesia and the Philippines

Childhood is socially constructed and fundamentally different from adulthood, continually varying (and evolving) across time, space/place, context, class, gender and cultures (Coster 2007; Editorial 1998; James and James 2004). Similarly, childhoods in the Philippines and Indonesia/Java are neither static nor uniform across and within the vast neighbouring archipelagos. In this section, we briefly outline the experiences of growing up in the study communities, making no claims to a universal understanding of childhood even for those living in the same country.

### Javanese childhood

From Geertz’s (1961) early influential work on socio-cultural life in Java, Javanese families are said to adopt a bilateral kinship system focusing on the nuclear family as the most important kinship unit with extended members offering aid and companionship when needed. Growing up, Javanese children are especially close to their ‘completely dependable’, nurturing and protective mothers who are often the main caregivers, disciplinarians and educators of the children (Geertz 1961, 106; Schröder-Butterfill and Kreager 2005).^1^ Mothers often make decisions on children’s behalf and are likely to retain custody in the event of a divorce. On the other hand, children maintain an affectionate and warm relationship with their fathers when they are between one and five years old,^2^ but grow distant though respectful thereafter (Geertz 1961; Jay 1969; Zeitlin et al. 1995).

Tracing childhood experiences of Javanese village children across three generations from the 1930s to the twenty-first century, White (2012) found that changes in schooling/education and consumerism were complicit in shaping children’s changing experiences of childhood.^3^ An education act introduced in 1952 and subsequently strictly enforced in 1984, made it compulsory for children to receive six years of education. Since 1993, children have to remain in school until age 15, though this is not strictly imposed. Remaining in school longer or achieving better qualifications, however, has not necessarily meant better prospects for the children due to the ‘“pushdown” effects of expanding access to education’ (White 2012, 94). Instead, children experience a prolonged childhood and adolescence, becoming increasingly dependent on their parents and older relatives for money amidst rising intergenerational tensions (White 2012).

At the same time, it is pertinent to note that the state has played a major part in influencing both gender roles and family life in Indonesia. Under Suharto’s New Order,^4^ gender relations were reshaped whereby fathers are portrayed as leaders in both public and private spheres, mothers as nurturers in the home, and children as only interested in playing (Agusta 2009; Blackburn 2004; Robinson 2000). Koning (2004, 234) argues that the ‘New Order gender ideology is far from being practised by the majority of its citizens’, manoeuvring women into narrowly defined roles while ignoring the multiple roles – including being the main breadwinner – many women often fulfil. As Brenner (2005, 116) noted, ‘fundamental contradictions’ are embedded within the gender ideologies of the Indonesian state whereby women are expected to be selfless pillars of their families while actively participating in the national economy. Women are expected to manage both their careers as well as their vocation as wives and mothers. In turn, the feminisation of labour migration has further challenged the New Order image of men as leaders and household heads.

### Filipino childhood

While there are few studies focusing on childhood in the Philippines, particularly from the perspectives of the Filipino children, studies on gender socialisation observe that boys tend to be playing outside to avoid being underfoot while girls are expected to take care of household chores (Añonuevo and Estospace 2002). Filipino children are schooled in specific gender differentiated roles at different life stages by close-knit family members and society through work (household tasks), play, school and the media (Liwag, de la Cruz, and Macapagal 1998; Medina 2001). Children – especially poorer rural children – are generally expected to help out. Gendered expectations in how they help become progressively pronounced from age ten to puberty. Girls are tasked to shadow their mothers in ‘caring activities’ including cooking, washing, cleaning, marketing and tending to younger siblings whereas boys are expected to assist in ‘strength-related activities’ such as agricultural work, repairs and maintenance (Medina 2001; Papa 2014). Girls live a comparatively restricted and protected life throughout their adolescence, and are basically taught the necessary skills to become gentle and modest wives and mothers. Progressively, girls are trained to be *tagasalo*, meaning a person who ‘takes over’ in the household, becoming responsible for reproductive functions such as childrearing, domestic work, and household management (Osteria 2011). Hence, their self-worth is intrinsically tied to caring work, being pleasing to and responsible for others (Añonuevo and Estospace 2002).

Unless there are no daughters in the family, boys are typically not asked to do feminine household tasks (Papa 2014). They have more freedom to stay out late and are taught breadwinning skills (Medina 2001). Becoming a man – according to the teenage boys in Lauer’s (2005) study – means being responsible, independent and capable of helping others, as well as being muscular, strong and athletic. Such manly qualities are sanctioned by the community, and boys aspire to achieve them in order to gain recognition and avoid ridicule.

Catholicism, family and extended kinship ties form the foundations of Filipino society (Yu 2015). Traditionally, Filipino families are ‘patriarchal in authority’ with the husbands’ breadwinning role (power) taking precedence over the wives’ position (Castro, Dado, and Tubesa 2008, 1; Pingol 2001). Though domesticity does not feature much during their childhood, boys are required to mature and become reliable immediately upon marrying or becoming fathers (Añonuevo and Estospace 2002). As household heads, Filipino men are imbued with ‘physical superiority’ (Osteria 2011, 14), and expected to be logical, confident, good providers, protectors, role models, ‘virile sex partners, firm and strong fathers’, the home’s handymen, with limited concerns over their children’s daily lives (Alampay and Jocson 2011; Medina 2001; Pingol 2001, 8).

However, globalisation, industrialisation and migration have since derailed existing gender stereotypes and family structures, affecting the country’s conventional family dynamics, parenting styles, and gender ideologies (Alampay and Jocson 2011; McKay 2012). Over time, the nucleation of families and rise of alternative marital arrangements have helped to elevate Filipino women’s position, bestowing them with greater autonomy and slowly eroding men’s authority at home (Osteria 2011). Society has also grown more accepting of mothers going to work, opening up further opportunities for women. Nonetheless, women are still expected to be the family’s problem solvers, bearing the brunt of any crisis. While men are now expected to contribute to the home by sharing in housework and childcare, women are still expected to pull in double duty by juggling work and domestic duties (Osteria 2011). Migrant women in particular encounter both praise and censure even as their sacrifices and earnings are celebrated while their absences are often denounced as an abandonment of their mothering duties (McKay 2005).

Overall, children are considered precious and important to Filipino parents for various reasons, leading them to make great sacrifices for their education and future (Medina 2001). Filipino parents, though nurturing and supportive, consider ‘children as extensions of themselves rather than individuals with their own rights’ (Osteria 2011, 18). Most of the descriptions of Filipino children are actually derived indirectly through accounts or behaviours of the adults toward children. Children are often overprotected and strictly disciplined but parents are increasingly complaining that they are becoming less ‘meek’ in this modernising world, answering back disrespectfully when admonished (Osteria 2011, 18). Parents learn to cope with these behavioural shifts by changing their parenting styles, becoming more open and permissive to relate to their charges better.

### Migration trends from Indonesia and the Philippines

Both Indonesia and the Philippines are archipelagos with long migration histories starting around the 1980s and 1970s respectively. Labour migration is an economic strategy actively pursued by the governments of both countries to promote development, cope with poverty as well as under- and un-employment. The increasing demand for Indonesian and Filipino female domestic workers to plug the care deficits in the homes of more developed economies has fuelled large numbers of female outmigration from both countries. Hence, remittances feature among the top revenue generators for both countries with Indonesia receiving at least US$8.9 billion (World Bank 2017) and the Philippines receiving some US$26.9 billion in 2016 (BSP 2018). In areas with high rates of international migration, the changing material landscape resulting from house-building projects testify to the flows of remittance monies. A summary of the migration trends and development indicators of the study sites is presented in Table 1.

**Table 1.** Summary of key information for Indonesia and the Philippines.

Country	Province	Migration (2015)			Top 3 Migrant Occupations	Top 3 Migrant Destinations	Total Remittance	School Enrollment (2014)	Literacy Rate (2013)
		Male	Female	Total	(2015)				
Indonesia	West Java^a^	12,728 (20%)	50,348 (80%)	63,076	- Domestic Worker (19,045) - Care Taker (17,433) - Operator (4,531)	- Taiwan (22,850) - Saudi Arabia (9,362) - UAE (4,636)	6 trillion Rupiahs^b^ (US$452 million)	- Elementary (97.60%)^c^ - Junior (79.30%)^c^ - High (56.48%)^c^	97.05%^d^
	East Java^a^	16,895 (35%)	31,417 (65%)	48,312	- Care Taker (12,477) - Domestic Worker (8,309) - Operator (5,621)	- Taiwan (23,286) - Malaysia (7,083) - Hong Kong (5,559)	5.6 trillion Rupiahs^b^ (US$429 million)	- Elementary (96.98%)^c^ - Junior (80.94%)^c^ - High (60.00%)^c^	91.12%^d^
Philippines	Calabarzon (Laguna)^e^	237,104 (56%)	186,002 (44%)	423,106	*Figures are based on Luzon’s total for Overseas Contract Workers*			- Primary (92.03%)^i^ - Secondary (68.60%)^i^	98.4%^j^
	Central Luzon (Bulacan)^e^	209,072 (57%)	154,609 (43%)	363,681	- Labourers & Unskilled Workers (445,556)^f^ - Services (237,452)^f^ - Operators (169,418)^f^	- Asia (1,151,242)^g^ - Europe (84,042)^g^ - Americas (68,034)^g^	7.4 trillion Pesos^h^ (US$1.6 trillion)	- Primary (95.64%)^i^ - Secondary (72.83%)^i^	98.2%^j^

Sources:

^a^BNP2TKI (2015).

^b^Susilo (2015).

^c^BPS Statistics Indonesia (2017a).

^d^BPS Statistics Indonesia (2017b).

^e^Philippine Statistics Authority (2015a).

^f^Philippine Statistics Authority (2015b).

^g^Philippine Statistics Authority (2015c).

^h^Philippine Statistics Authority (2015d).

^i^Albert and Raymundo (2016).

^j^Philippine Statistics Authority (2015e).

## Methodology

The data for this paper is drawn from CHAMPSEA, a project investigating the impacts of parental migration on various aspects of children’s physical, psychological, social, emotional and educational well-being and health in Southeast Asia (Graham and Yeoh 2013). The quantitative data is derived from surveys conducted in 2008 with roughly equal proportions of transnational and non-migrant households containing children aged 9–11 in East and West Java, Indonesia (*n* = 513 households), and Laguna and Bulacan in the Philippines (*n* = 500 households). Eligible households were intact, heterosexual families, with at least one child in the target age group (either 3–5 years or 9–11 years).^5^

Subsequently, follow-up qualitative in-depth interviews with selected surveyed households were conducted in two phases; first, with 26 Indonesian (from East Java) and 28 Filipino carers (from Laguna), as well as semi-structured interviews with 32 children aged 9–11^6^ (16 per country) in 2009, and second, interviews with 20 households comprising returned-migrants, left-behind carers and children between 2009 and 2012. Interviewers were sensitive to children’s moods, and started the interviews only after obtaining both informed parental consent and children’s assent. To make the children feel safe, confident and comfortable, interviews began with ice-breaking activities such as the ‘Protection Umbrella’ (see Beazley et al. 2005). Children were also encouraged to express their views freely and told that there were no right or wrong answers. Interviews were mainly conducted in native languages and translated into English for analysis. Pseudonyms are used in this paper to ensure respondents’ anonymity.

## Children’s sensitivity to parental absence

Migrating for the sake of the children’s future/education was the main reason why many Indonesian and Filipino parents in this study undertook the migration gamble.^7^ Their intentions did not go unnoticed as a larger proportion (52%) of the primary-school aged children (among the 78% who were aware of the reason their parent(s) migrated) answered ‘my education’ as the main reason their father and/or mother migrated (Lam and Yeoh 2018b).^8^ The majority (86%) were also able to recall and identify who told them about their parents’ migration. When asked to describe their feelings about their parents’ departure, the majority (71%) chose the survey answer ‘sad, I miss him/her’ (Table 2). There was no significant difference in responses according to gender as both left-behind boys and girls expressed missing their absent parent, be they a migrant father or mother. Interestingly, migration may in fact have become a way of life for some children, suggested by those (12%) who stated that they had no feelings about their father/mother’s migration.

**Table 2.** Feelings of Indonesian and Filipino children aged 9–11 about their father/mother’s migration (percentage).

Feelings about father/mother’s migration	Indonesia		Philippines		Total
	Father’s	Mother’s	Father’s	Mother’s	
*N*	115	186	189	83	573
Sad, I miss him/her	62	66	76	86	71
Nothing/like usual	20	15	7	6	12
Both sad and happy	11	9	10	2	9
Happy because s/he gets me toys/presents	7	10	4	4	7
Other	0	1	3	2	2

Source: CHAMPSEA survey data.

Nonetheless, how migration may have affected the children was not always clear as they express their sadness differently. Some children showed their pain outwardly, others appeared impassive or indifferent whilst actually feeling ‘broken hearted’. In particular, the quantitative survey data revealed that Indonesian and Filipino children of mother-migrants were less likely to be happy compared to those in non-migrant households (Graham et al. 2012). Based on the general happiness indicator, 85% of children in non-migrant households stated that they were very happy or happy, compared to 78% in father-migrant households, 75% in mother-migrant households and 79% from both-parent-migrant households (*p* < .001) (Jordan and Graham 2012).

## Influencing parental migration decision-making

### Children’s lack of voice

Ironically, given children’s educational futures were parents’ main consideration for migration, children were often invisible in the migration decision-making process (Huang and Yeoh 2011). In interviews, left-behind children reflected that they had no say in deciding if their father or mother should migrate and/or who should migrate. Migration was solely their parents’ decision, and most tended to inform their children personally or through other family members prior to their imminent departure. Very few parents sought their young children’s opinions. A Filipino mother described her reasoning:

At that time, my husband did not have work. … it was very painful for me to leave my children, … but … I’ve got financial problems and I know that for me, this is the only chance I can support the study of my children so I grabbed it. … my husband was not allowing me to go. He said don’t go, we must be together. … But I don’t want them to starve. I know my children like to study. So I ignored him. But I told my kids, especially my eldest son … [that] I have to go to Saudi Arabia because I want to help them in their studies … my youngest child was … very small at that time, so she doesn’t know … [that] she’s very lucky because of the sacrifices that her mom made. (Amanda, 44, Filipino-returned-mother-migrant)

### Stoicism for the common good

Despite their youthfulness, children gradually adapted to their family’s situation by taking the first steps towards understanding the reasons for their parents’ migration. Resilience was usually articulated by drawing on the discourse that migration was a family livelihood strategy where the family worked hard together so that children could succeed in life. Some started to grasp the meaning of sacrifice, learning to hide their sadness behind the notion that migration was for the greater good of the family. Kelvin (11, Filipino) calmly reasoned:

I think I was 9 or 10 years old [when she left]. … [When] they told me that my mother was going away for us so [that] we would have a nicer house, nicer life and even better studies, I told them if it is really needed, it’s ok with me … I told them that … but deep inside, I felt so sad because not having a mother is so difficult, especially for someone like me who was going to be a teenager soon.

### Children’s ways of voicing their wishes

As children grew older, many became less accepting of their migrant parents’ absence and began asserting their agency by employing either direct or indirect measures to persuade their parent to return home. Some nagged their parent to return during their long-distance conversations. Using her grandfather’s sickness as a reason, Jade (10, Filipino) commanded her mother over the telephone, ‘Come home! Grandfather is sick!’, leaving her mother, Veronica (34, Filipino-returned-mother-migrant), in tears: ‘She really wants me to go home. She misses me. “Come home!” I cry because I want to go home but I can’t. It’s very hard … [but I tell her] yes, I will soon. *Malapit na* [very soon]’.

Yuda (11, Indonesian) who had successfully engineered his mother’s return was able to say confidently, ‘I did [ask her to come home]. *Ibu* [Mother], please come back soon. [I asked her to return] because I felt alone at home’. His cajoling worked as Hanif, his 38 year-old father-carer, affirmed: ‘Her contract was finished. [She did not renew]. She has children … who wanted her to come home’.

Just as migrants regarded the ‘remittances of money, gifts, and services … as “transnational acts of recognition”’, children’s act of requesting gifts bore greater significance than simply the articulation of material wants (Yeoh et al. 2013, 441). Left-behind children tried to exert their agency over their migrant parents to ensure that they remained in their absent parents’ thoughts, or to secure their parents’ commitment to return home, even if it was only for a short visit. For instance, while Berlian (11, Indonesian) was aware that her father was unlikely to return home permanently, she strategically tagged his visit to a gift. ‘[My father] calls every day. I wanted him to come back [but I did not tell him so]. Never. [But I asked him], “Dad, come home quickly [for a visit] and will you buy me a doll?”’

When direct methods failed, children were still able to exercise their agency by turning to subtler, indirect measures to get their message across and influence their migrant parents’ behaviour. Perwira’s (10, Indonesian) mother returned as soon as her contract was completed primarily because of the deterioration of his eating and studying habits while she was away. His mother, Melati (32, Indonesian-returned-mother-migrant), explained:

Before I left, he ate independently. After I left, he cannot eat independently and just ate small portions. … He got worse [in his studies while I was away] because only my husband was looking after him … It’s important for him to take a nap in the afternoon after he gets back from school and has eaten. Now that I’m home, I try to feed him, ask him to eat more and accompany him. Now, he’s better.

Children with lengthy experiences of parental migration had mixed feelings about their parents’ absence. Many were quick to cite the positive material benefits gained from migration, including studying in a better school, having better toys and wearing nicer clothes and shoes. However, they were fully aware that these perks came at the price of a divided family and the pain of missing their absent parent. When asked if they would be willing to see their parent, either father or mother, migrate again, many children’s initial reaction was a strong ‘no’, with very specific reasons such as:

When she’s not around, I don’t have anyone to share my stories with. [The family is happiest when everybody is together]. (Marianne, 9, Filipino)

There was a desire for the family to remain together regardless of their economic situation and this was further emphasised when children were asked if they would personally like to migrate when they are older. While some of the children, especially Filipino girls, were enthusiastic in becoming overseas migrant workers, they immediately made clear that they would not do so if they had a family of their own.

If it is needed [I will work overseas like Mommy]. No [I will not leave children behind]. No [I will not let my husband go overseas]. I want my family to stay together. (Magdalene, 9, Filipino)

Repeat migrations were common in Indonesia and the Philippines, but the decision to leave did not come any easier for migrants or their families during subsequent moves, especially as children grew older and their opinions mattered more. Parents sometimes postponed or gave up their migratory plans for them, as Melati related: ‘I was asked to work there again, but my children did not allow me, so I did not leave [laughs]’. Nonetheless, despite their negotiations and concerns, the need ‘to earn some money again’ or ‘to pay school fees’ may ultimately prevail and force families to undergo the migration and separation process all over again.

In attempting to provide their children with the best, some families seem to become entangled within a never-ending migration cycle. The children, while enjoying the fruits of their parents’ labour, do not appear to fully appreciate the necessity of familial separation as a livelihood strategy.

## Re-weaving the web of care: coping with parental migration

Given that children’s happiness and childhood are not just dependent on who migrates but also on the care they receive, this article now examines children’s varied reactions and ways of coping with the ensuing care arrangements during their migrant parents’ absence. Their responses and the ways in which they shift from ‘dependence and often passivity … to actively chart specific strategies to cope with new environments’ (Huang and Yeoh 2011, 396) reveal their agency, resilience and creativity^9^ in influencing caring practices in their migrant family.

### Acquiescence to father’s absence

Caregiving arrangements in CHAMPSEA’s families tended to remain fairly stable when fathers migrated with the aim of continuing their breadwinning role, as mothers retained their role as caregivers (Lam and Yeoh 2018a). Children of mother-carers appeared contented to accept this traditional gendered care arrangement, citing reasons such as ‘Because Daddy isn’t here. Besides, we are already used to having Mommy taking care of us … I’m closer to Mommy because it’s Mommy who is always with us’ (Gladys, 10, Filipino).

While children might appear passive in accepting this long-standing arrangement of having mothers as their main caregivers, their acquiescence stemmed from their positive reception to the ways (‘her love/care’, ‘she’s supportive’, ‘she’s patient’, ‘she defends us’, and ‘she feeds me and raises me’) their mothers have cared for them. They differentiated between the ways their mothers and fathers care for them, as seen in Yuni’s (10, Indonesian) reflection:

If there is something I don’t understand, she [mother] will teach me. She won’t scold me … [With] mother, she gives more attention. The way she teaches us is different. [Father] does not teach us, [but] just asks us to do it.

Hence, they are ‘happy’ and actively chose to remain under their mother’s care since ‘We’re happy as a family’ (Justin, 9, Filipino), ‘I’m glad [mother cares for me] because it’s like when I’m with father’ (Setyo, 9, Indonesian), and ‘There is no one else. It’s just Mama, really’ (Candice, 11, Filipino). Trusting in their mother’s protection, they could turn to her when they encountered problems.

Overall, there were no apparent major disruptions at home, apart from mothers imposing stricter disciplinary measures while expecting children to share housework as part of their strategy of coping with added household responsibilities during their husbands’ absence. However, evidence from multivariate models generated from the CHAMPSEA quantitative dataset showed that emotional disorders were more likely among Indonesian children in father-migrant/mother-caregiver households as compared to those living with both parents (Graham and Jordan 2011). Conversely, there were no significant differences in emotional symptoms among Filipino children living in different types of households when confounders were included. The selected child interviewees did not indicate that anything was amiss and further research examining the contextual factors such as cultural norms relating to women’s societal roles and intimate relations within both household types may be necessary to explain this seemingly counter-intuitive finding (Graham and Jordan 2011).

### Resilient navigators in mothers’ absence

The migration of mothers, by contrast, revealed more diversified care arrangements involving a web of carers, although in the majority of mother-migrant households, fathers constitute the most important group of primary carers for children (68% in Indonesia and 60% in the Philippines). Nevertheless, even when the main caregiver was identified as a specific person such as the father or grandmother, many children – by their own accounts – continued to experience being cared for by different members of the family (and even non-relatives such as domestic workers), particularly siblings, in different capacities. For example, Joel (9, Filipino) explained that ‘father feeds me [and] buys me medicine, brother helps me with my schoolwork’ while Nardi (11, Indonesian) said, ‘grandmother woke me, napped with me, cooks for me. Father irons the clothes, buys me school stuff and helps me with schoolwork’. Children were very aware of the different persons catering to their needs and were able to identify and navigate around the diversity of care in their daily lives.

Although children in mother-migrant households were generally accepting of their care arrangements, many were more vocal about their fractured family circumstances. They tended to praise the efforts of their father-carers while lamenting about the incompleteness of their family. For instance, Shirot’s (11, Indonesian) web of care comprised his left-behind father, sister and himself working together to ensure that his daily needs for food, clothing and schooling were met. Despite his father’s seemingly minor contribution to the physical care of the household, Shirot was still appreciative of his father’s emotional and financial caregiving efforts. He pointed out that his father was ‘clever in making people happy. He is a proper head of the family. … He always gives me sufficient pocket money. Sometimes my friends do not get any pocket money’. Even though Shirot wished to be cared for by both parents, feeling that his ‘family is incomplete’, he sought to make the best of his current familial care arrangement.

Similarly, Carl (9, Filipino) understood that his mother’s migration was essential in helping the family acquire what they ‘need’ whilst appreciating his father’s good care.^10^ However, he very much preferred to be cared for by his absent mother:

[Papa] feeds me, teaches me. He plays with me. He helps me when there are things I don’t understand. Yes, [I like how Papa takes care of me] when he plays with me. None, [there’s nothing I dislike about the way he cares for me. But, if given a choice, I would still choose] Mommy. When I want to go to … Jollibee [a popular Filipino fast-food chain], we go there [together]. [With Papa] there’s no time.

Carl felt ‘sad’ when he talks to his mother over the phone ‘once in a while’. His mother tried to bridge the distance between them but he wished for her return and told her accordingly. Unable to alter the family caregiving arrangements, it was clear that he had started trying to effect change by voicing his feelings.

Overall, father-carers in this study appeared to be managing well.^11^ Contrary to popular belief, Filipino children left in their father’s care appeared to be less likely to suffer serious conduct problems compared to children living with both parents. There were no significant differences in conduct problems when child age and gender were accounted for among Indonesian children living in different household types. These more-positive-than-expected findings relating to father-carers have been substantiated by accounts of how fathers were often supported by a strong network of ‘other mothers’ providing intimate care for their children (Lam and Yeoh 2014).

Nonetheless, many children with mother-migrants such as Shirot and Carl retained gendered expectations of care and preferred their mothers to be home caring for them. Having initially not been availed substitute maternal care arrangements, they gently navigated around their transnationally-split family situation to rework the web of care to suit their needs.

### Coping with gender role conflict

As changes in gender roles become more entrenched within their households, some children appeared conflicted toward their rewoven web of care. Eunice (11, Filipino) was cared for by her father Enrico (39) while her mother (with whom she is in daily contact via webcam) worked overseas. She was cognisant of his dedication and direct involvement in her day-to-day care but remained strong in her views that he should not be doing ‘women’s work’:

He’s the one who cooks breakfast for us, and when I’m sick he’s the one who takes care of me. He’s the one who launders the clothes. He attends meetings. Sometimes, during enrolment, he’s the one who accompanies me. He’s okay but it is different if the mother is the one caring. Because the mother is of course the light of the home. … Papa … should be doing manly work right? Mama is the one doing the man’s work.

Eunice’s grandmother helped her father with caregiving, especially when Eunice was sick. She felt closest to her maternal grandmother, demonstrating her preference by sleeping at her grandmother’s. Her father observed: ‘She’s there almost every day’. Nonetheless, Eunice – when questioned if she would confide in her grandmother when she felt sad or had a problem – replied: ‘No. I’m embarrassed’. She did not view her father as a source of support in such circumstances either. Though she was amenable to seeking her father’s help with schoolwork, Eunice coped with sad moments by keeping to herself: ‘I don’t go to anyone … I just keep it a secret’. She was in the habit of writing her inner thoughts in her diary.

Eunice’s narrative illustrates how some children cope as they grew older in the prolonged absence of their mothers. Becoming more vocal about the changes in gendered performances of care and breadwinning in her family, Eunice managed to navigate around the available care arrangements to fulfil her needs for immediate ‘motherly care’ in her mother’s absence, although she was unable to exert any significant agency in terms of the family’s migration strategy and withheld her emotions from her *in situ* carers.

While the majority of the children held on to gender ideals that their mothers should be their carers in the ‘natural’ order of things, there were signs of shifts over time, particularly in cases when mothers had been away for most of their growing up years. Awuna (11, Indonesian) had grown somewhat immune to her mother’s repeated absence over the course of her childhood, claiming that she felt ‘just okay, I did not cry’ when her mother left to begin yet another contract two years ago. She felt that she had flourished under her father’s (Abadi, 42) care, as affirmed by achieving third place in her school. She was eager to gain her father’s approval and had a high regard for his care,

[My mother] has gone abroad several times. Always to Taiwan but in different workplaces. Mother [is more suited to be the migrant]. I have stayed with my father for a long time … [and he] can take care of the house. If my father left, my mother would have difficulties. She is unable to cover all the house chores that father is able to.

Ironically, though her mother had been working as a domestic helper in Taiwan for the past six years, Awuna did not see her as capable of handling the chores at their own home as compared to her father. Over time, Awuna had in fact come to accept a reversal in the traditional gender notions of care.

### Rejection

The complete rejection of a migrant-parent’s care is one possible extreme outcome of the modification of family care roles. While most children interviewed expressed happiness and excitement at being able to speak to their migrant parent over the phone, and were receptive of the various efforts they made to care for them despite physical separation, not all were keen to maintain a relationship with the absent parent. Sira (9, Indonesian) for example, rejected his mother-migrant’s attempts to care by refusing to answer her calls. He stated that he did not miss his mother and ‘never’ tried calling her. He preferred his primary caregiver, his maternal aunt Hanaya (38). His left-behind father and mother-migrant both tried to persuade him otherwise. His mother often cried, wanting to hear her son’s voice. Sira’s father added: ‘It is different with the younger child who asks “let me talk to mother”’ when he calls his wife.

Upon further probing, Sira revealed his reason behind the strained mother-son relationship, ‘She’s mean. [She gets angry with me] because I don’t finish eating my meals’. Avoiding his mother’s calls was a method adopted by Sira to escape her discipline. The estrangement reached a point where the migrant had to threaten withholding gifts if Sira continued refusing her calls. Migration can both alienate children from parents (Poeze 2019) and amplify existing tensions in the pre-existing web of care.

## Resilience and self-reliance of left-behind children

The CHAMPSEA study, along with previous research (see Graham et al. 2012; Hoang et al. 2015; Pantea 2012), reveals the diversity of negotiated care arrangements and care experiences for left-behind children in their home spaces. The influence of existing gender-normative discourses suggests that children living in transnational households experience mother-care, father-care, and care from ‘other mothers’ differently, and that this may affect their assessments of their own well-being. The children’s narratives reveal the stickiness of views of traditional gendered caring roles. More importantly, the narratives show that children are learning to exert their voices and autonomy in the family’s migration and caregiving decisions as they mature. Despite still being largely dependent on adults, children aged 9–11 were gradually moving out of passivity to actively craft strategies to cope with their families’ migration circumstances.

Thus, the changing autonomy of left-behind children as they grow up and their potential role in their own web of care should be taken into account. As Asis (2006) noted, children often have more empowering opportunities to develop and grow outside of their migrant parents’ constant and restrictive control. At the same time, Majorano et al. (2015, 3444) found that parent-related loneliness or a gradual detachment from parents, may be a ‘healthy’ experience for adolescents in their transition into adulthood. Given their family situation, many left-behind children would have also been trained by left-behind carers to be independent in caring for themselves. Grandmother-carer Alicia (62, Filipino) espoused this philosophy:

During schooldays, at night I already prepare the clothes, socks, shoes that she [her grandchild] will be wearing. … I am starting to train her … she has to learn how to do it herself because I won’t be around for her all the time. The time will come when I will get sick, then who will do those things for her. I told her to learn how to do the things I’ve been doing for her … I told her that she should learn those things so that when her mom comes back, she will be happy to know that her daughter can take care of herself.

Most children want to exert more independence in caring for themselves as they grew older. They exhibit what Katz (2004) terms as ‘resilience’, rationalising their circumstances and coping with fractured family arrangements or lapses in care. When their carers were busy, left-behind children were able to independently prepare their own lunches, launder their own clothes and occupy themselves at home after school.

A father’s absence may influence older boys who feel they have to grow up quickly to fill their father’s shoes. Vincent (10, Filipino) had become increasingly independent during his father’s absence, and had started doing many things by and for himself. He confided: ‘for example, when I depend on my mother for cooking [breakfast], I always go to school a little bit late. [So I do it by myself]. I’m kind of used to it [waking up by myself]’. He felt embarrassed if his mother fetched him from school. This had become a point of contention between them, requiring his migrant-father to step in and mediate. Perhaps his wish to exert his independence from an early age stemmed from his ‘burden’ at being told by everyone around him that he is now the ‘man of the house’. A robbery at his house further heightened his sense of responsibility that he needed to quickly ‘become a man’ in order to protect his mother and younger brother in his father’s absence. Interestingly, girls with mother-migrants in this study did not express the same imperative to become the ‘woman’ of the house, possibly because many turned to older sisters or other family members (including fathers) for help.

Even if they cannot dictate their parents’ migration trajectories, several left-behind children want autonomy to make their own decisions regarding their present and future lives. Some had decided on the school they wished to attend and the courses they wanted to take (sometimes in opposition to their parents’ wishes). Yuni wanted to take additional tuition classes with her friends and actively asked her mother-carer for money to do so. Eric would have already transferred his son to a better school but Carl refused as he did not know anyone at the new school. To her parents’ dismay, Eunice refused to be enrolled in the school’s pilot section (the best class), preferring to stay in her existing section where she was the top student and practically guaranteed a medal. Despite repeated cajoling and encouragement from her parents, Eunice remained firm and her mother gave in, saying ‘Okay, let it be, just do well’.

Although most children largely accepted the care arrangements planned for them, some – especially those left with non-parental carers – had a greater degree of freedom and took a more proactive approach in deciding who their carers should be from the outset. An example is Claire (10, Filipino), who decided to exercise her growing sense of autonomy in her mother’s absence by arranging her own care and going directly to the carer of her own choosing:

[When Mommy left] I lived temporarily with my aunt, the sister of my father because I was the only girl [here]. After 6 months, I came home. Because my father and brothers were here. [Living with my aunt and returning home was all] my decision.

Her mother, Jenna (31), had initially entrusted Claire’s care to her own mother (Bella [54], Claire’s maternal grandmother) as the main carer and her husband as the secondary carer since he was working. However, she was worried and entreated her siblings as well as eldest son to look after Claire. Claire resisted her parents’ plans and chose instead to spend six months with her paternal aunt (someone not of her mother’s choosing). Even after she returned home, Claire tended to prefer the care of her eldest brother, who ‘advises’ and ‘tells’ her what to do and who she can consult when she has problems. While her grandmother was designated her primary carer, Claire considered her mainly as someone who just ‘checked on us if we were home’.

Intriguingly, some left-behind children seemed to revert to more childlike behaviour when the migrant parent returned. Dumadi (10, Indonesian) and his returned-mother-migrant Bethari (33) both agreed that he had become more *manja* (childlike) after his mother returned home. Jade (10, Filipino) wanted her mother to carry her ‘like a baby’ even though she had grown much bigger in the 4-year interval. Despite the fact that they were well able to care for themselves, they preferred their returned parent to do everything for them and would stick closely by their side, exerting their agency in reclaiming their returned-migrant parents’ care and re-stitching familial relations (Lam and Yeoh 2018b). Their behaviour possibly compensated for lost childhood time with their formerly absent parent. This was a situation that many returning parents were pleased to encourage as a means of re-bonding with their children.

## Conclusion

While Javanese and Filipino childhoods are culturally and contextually different, the children in this study share the commonality of growing up in gendered migrant-sending communities where parents often migrated as solo contract workers under the prevailing temporary migration regime in Southeast Asia. Our study demonstrates that children in migrant families positioned within a web of home care linked to their migrant parents’ absence are similarly and simultaneously powerful and powerless (Dreby 2007; Hoang et al. 2015). The left-behind children’s accounts revealed how they grew from ‘passivity’ or ‘incapacity’ into increasingly active adolescent agents within the context of their care environments. As Ortner (2006) argues, conditions of agency relate to context and the intention and ability to effect change. From the children’s own voices and actions, and those of their carers’ and returned-parents’, valuable insights into children’s spoken and unspoken memories, experiences, reasoning and behaviour emerge in the light of parental migration.

Javanese and Filipino children, seemingly passive in the presence of adults, are nonetheless aware and reflective of their situation. Even when they exhibit a ‘diminished form of agency’ (Collins and Tymko 2015, 77) in having little control over their parents’ migration decision-making, children from both countries are forming their own autonomous opinions of their parents’ absence and care arrangements, and developing and articulating their agency through examples of acquiescence, resilience, and rejection that may influence their parents’ decisions about migration. Over time, they learn to navigate their way into becoming social actors within their families, whether in decisions pertaining to care arrangements or their own education. Situated in a position relative to other persons of differing ages and power, children are constantly experimenting, adjusting, resisting and reworking plans independently. In response, adults may also alter their plans to cater to their children’s wishes or negotiate with them to reach a compromise.

Children’s agency need not reveal itself in a ‘big’ way or bring about ‘major’ changes (James and James 2004). However, children’s simple wishes or more forceful demands often have profound, intended as well as unintended consequences for themselves and those around them. Hence, the ensuing interactions between children and adults make ‘a difference – to a relationship, a decision, to the workings of a set of social assumptions or constraints’ (Mayall 2002, 21). Similarly, the narratives recounted by left-behind children in this study reveal their direct and subconscious actions, affecting their family’s care arrangements, performances of gender roles and even their parents’ migration decision-making. These reactions do not form a linear progression in which a child proceeds from one stage to the next. They may in fact be circular, regressive or multi-directional as children constantly experience new information and feelings. While we have not undertaken a comparison between Javanese and Filipino children’s reactions to parental migration in this paper, we note a similar tendency as the children grew older, becoming bolder and more confident, and unafraid to express their feelings to their left-behind carer, migrant-parent or even a stranger in the form of the interviewer.

When confronted with the multiple disruptions and changes in gender and care ideologies in their lives caused by migration, left-behind children’s reactions range over a wide spectrum of both positive and negative behaviour. Despite encountering changes in gender roles within their own families, many children express comfort in sticking to traditional gender ideologies where the main responsibilities of breadwinning and caregiving were separately borne by the fathers and mothers respectively. However, children are also flexible in adjusting their mind-sets, often temporarily, to accept the alternative arrangement for the sake of their family. Whether the winds of change blowing through the region’s migrant-sending villages are experiencing ideological change in gender roles across the generations needs to be further explored.

## Notes

1 Mothers engaged in paid work outside the home may briefly leave children with other female relatives.

2 Fathers played with, bathed, fed and cuddled their children during this period.

3 Unfortunately, the study did not mention how migration may have affected childhoods.

4 The ‘New Order’, a term coined by the second Indonesian President, Suharto, characterised his regime when he came to power in 1996. The regime strengthened the political role of the military, and led to the bureaucratisation and corporatisation of social and political organisations. It ended in 1998 when the Suharto government was toppled by pro-democracy movements.

5 A key limitation in CHAMPSEA is the exclusion of other family types by only sampling intact families.

6 Children’s ages were first captured in 2008 and have grown over the course of the study.

7 Other responses (in descending order) include, general family care/subsistence; household projects; no jobs/work here; salaries are too low here. Also, the primary reason Indonesian mothers migrated was for general family care/subsistence, followed by their child’s future/education.

8 The same proportion of Indonesian children chose ‘for my/our education’ and ‘general family/subsistence’ as the main reason their fathers migrated. Other responses (in descending order) include: general family care/subsistence; household projects; no jobs/work here; other/don't know.

9 The idea is derived from Katz’s (2004) concept of children’s agency expressed through their everyday responses of resilience, reworking and resistance.

10 His father, Eric (38), was personally aware that he was unable to monitor Carl as closely as his mother, which affected Carl’s performance in school negatively.

11 Similarly, earlier analysis by Graham and Jordan (2011) indicated that primary-school-aged Indonesian children cared for by fathers did not suffer a care deficit in respect of their psychological well-being when compared with children in non-migrant households.
